# A Longitudinal Study on the Relationship between Physical Education and Social Health

**DOI:** 10.3390/healthcare9091134

**Published:** 2021-08-31

**Authors:** Hanguk Cheon, Seijun Lim

**Affiliations:** 1 Department of Physical Education, BaeMyeong High School, Seoul 05598, Korea; davidcheon@daum.net; 2 College of Physical Education, Kyung Hee University, Yongin-si 10315, Korea

**Keywords:** Korean adolescents, effects of physical education, social health, social relationship, parental relationship, peer relationship, relationship with teachers, sense of community, reciprocal causation, autoregressive cross-lagged model

## Abstract

This study aimed to examine whether school physical education (PE) promotes students’ social health using a longitudinal design. To this end, data from 1979 students from a 4th grade cohort, established by the Korean Children and Youth Panel Survey with data collected from 2010 to 2016, who participated in all of the 7th grade (2013), 8th grade (2014), and 9th grade (2015) surveys were analyzed. We used autoregressive cross-lagged structural equation modeling conducted with AMOS 23.0 to examine the longitudinal causality among the factors. The following results were obtained. First, there was reciprocal causation between PE and social health (PE→SR, β = 0.099, β = 0.100; SR→PE; β = 0.207, β = 0.226). Second, PE did not influence sense of community, whereas the latter had a negative effect on PE (β = −0.078, β = −0.077). Third, social relationships influenced the sense of community (β = 0.248, β = 0.266). Based on these findings, we suggest the need for a system that enables students to frequently monitor their performance. This includes implementing a program to improve social relationships to enhance the quality of PE participation, designing programs that foster a sense of community in PE in Korean middle schools, and structuring PE programs that consider the hierarchy between social relationships and a sense of community.

## 1. Introduction

Traditionally, a major research topic among physical education (PE) scholars has been substantiating its effectiveness. As a result of such endeavors, school PE is known to be effective in promoting students’ physical, lifestyle, affective, social, and cognitive development [1,2]. These endeavors have led to mandatory implementation of PE in the school curriculum in 97% of countries worldwide [3]. This is because understanding and improving health among students through PE directly leads to better health of the future members of society [4].

Many PE scholars have argued that school PE promotes students’ mental and social health, but some are pessimistic [5]. There is doubt as to whether there is a definitive causal relationship between PE participation and mental and social health. To substantiate such causation, three conditions must be met: covariate analysis, temporal priority, and elimination of external explanations [6]. However, the pessimism lies in whether the evidence accrued by PE scholars satisfies all three conditions. For instance, regarding the structural relationships among adolescents’ exercise frequency, mental health, school satisfaction, and happiness, it has been argued that exercise frequency has a causal relationship with mental health, school satisfaction, and happiness [7]. However, there were no specific data on whether exercise frequency has temporal priority for mental health, school satisfaction, or happiness. This is occasionally evident in other studies as well [8]. Furthermore, experimental studies are necessary to eliminate external explanations, but it is difficult to conduct large-scale studies using this type of study design. In other words, the causation pertaining to the effectiveness of PE has generally been argued without sufficient evidence to support it. While some studies have applied research methodologies to analyze causation in light of these concerns, they have indirectly acknowledged certain limitations, as evident by their use of the term “correlation” compared to “causation” [9].

The failure to prove clear causation is attributable to the lack of large-scale, long-term assessments [10]. Furthermore, generalizable conclusions could not be drawn because of the lack of reliable monitoring and evaluation of student development [2]. Therefore, stronger evidence is required to support the argument that advocates for the potential benefits of PE [5]. With insufficient scientific evidence supporting the effects of PE, precautions should be taken against unproven facts being utilized as evidence of the effectiveness of PE [11]. For these reasons, Opstoel et al. [5] have recommended longitudinal study designs in the research and development of PE classes, based on the notion that generalized evidence supporting the effects of PE can be acquired only by garnering evidence showing that PE facilitates students’ development through a long-term follow-up [10].

Therefore, this study conducted a large-scale, long-term follow-up of the relationship between PE and social health. Among the physical, mental, and social effects of PE, we focused on social aspects. More specifically, we longitudinally explored whether there is a causal relationship between accomplishment in PE classes, social relationships (parents, peers, and teachers), and a sense of community. The ultimate aim was to address previous limitations and obtain evidence supporting the relationship between PE and social health (social relationships and sense of community).

### Study Questions and Study Models

The following study questions were established to accomplish our objectives:

1. How does the relationship between PE class achievement and social relationships develop over time?
2. How does the relationship between PE class achievement and a sense of community emerge over time?
3. How does the relationship between social relationships and a sense of community emerge over time?

**Figure 1:**
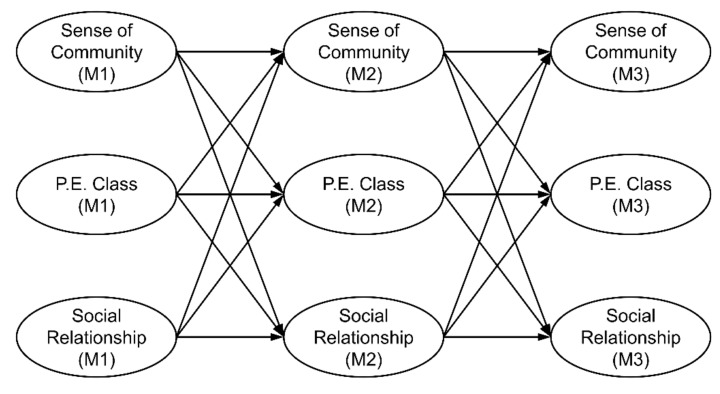
Research Model.

To answer these questions, we established an autoregressive cross-lagged model, as shown in Figure 1. The causal relationship was examined among PE achievement, social relationships (with their parents, peers, teachers), and sense of community in the 7th, 8th, and 9th grades. 

The study model was established based on the following literature: (i) PE and social relationships, such as how exercise and sports foster an environment promoting social relationships, how adolescents undergo social training in such environments [12,13,14], and improvements in social relationships [9,15,16] based on social capital and social skills learned during social training [17]; and (ii) PE and sense of community in relation to changes in social connections that trigger psychological changes [18] and how adolescents come to identify themselves as a member of a community [9,19,20,21]. The research model covered three time points according to middle school grades. At each time point, the students’ levels of physical achievement, social relationships, and sense of community were compared with those at the subsequent time point.

## 2. Materials and Methods

### 2.1. Study Data and Participants

In this study, we used data from the Korea Children and Youth Panel Survey (KCYPS 2010) conducted by the National Youth Policy Institute from 2010 to 2016 to examine adolescents’ personal growth and developmental environment over time. 

KCYPS 2010 was based on 1st and 4th graders in elementary schools and 1st graders in middle school in Korea, as of 2010. A total of 7071 adolescents from three cohorts extracted using the multi-stratified colony sampling method were followed up for 7 years from 2010 to 2016. The target sample was set at 6600, and the number of samples was allocated to each region in proportion to the number of students per grade level and schooling level in 16 metropolitan cities and provinces in 2010. The number of schools to be surveyed was calculated by examining all the students in each class, one class per school, and expecting a survey success rate of 80%. The target schools were selected based on the probability proportional sampling method (PPS) for each of the 27 colonies that were extracted by crossing 16 metropolitan cities/provinces and city scales (large cities/small towns/gun areas). The sample class was randomly selected after checking the information on the number of classes in the corresponding grade for each school and the number of students per class. According to this procedure, 2378 students from 95 classes across the country were selected as study subjects for the 4th grade cohort of elementary school.

These data were collected from a 4th grade cohort in 2010 until they reached the 10th grade in 2016. A total of 2378 4th graders participated in 2010, and 1979 (83.2%) completed the cohort data collection in 2016. The data in this study comprised responses from 2092 7th graders (1110 boys and 992 girls), 2070 8th graders (1089 boys and 981 girls), and 2061 9th graders (1091 boys and 970 girls). We ultimately analyzed the data from 1979 students who participated in all the three time-points. All schools in Korea must comply with the national level curriculum. Middle schools, the subject of this study, provide physical education classes for 3 h per week for all students. The study was approved by the Public Institutional Bioethics Committee of the Ministry of Health and Welfare, Korea (P01-202105-22-011).

### 2.2. Instruments

From the 4th grader panel data, we used data for PE achievement (2 items), relationship with parents (4 items), peer relationships (4 items), relationship with teachers (4 items), and sense of community (4 items). For PE achievement, grades were rated on an 8-point scale, while subjective accomplishment was rated on a 5-point scale. Additionally, social relationships (with parents, peers, and teachers) and sense of community were rated on a 4-point scale. The details of the items are presented in the Appendix A. 

### 2.3. Data Analysis

Data were analyzed using IBM SPSS software 23.0(IBM Corp., Armonk, NY, USA). The mean, standard deviation, skewness, kurtosis, and reliability (Cronbach’s alpha) of each item were computed using SPSS 23.0. Normality of the data was tested with skewness and kurtosis, and the reliability of the items was determined by Cronbach’s alpha. Additionally, correlation analysis (Pearson’s correlation coefficient), model fit (comparative fit index (CFI), root mean square error of approximation (RMSEA), standardized root mean square residual (SRMR)), and path analyses among the variables were conducted using AMOS 23.0 (Build 1607, IBM Corp., Armonk, NY, USA). 

## 3. Results

### 3.1. Descriptive Statistics

The absolute values of skewness and kurtosis of the responses at each time-point were less than one and two, respectively. This satisfies the requirement for normal distribution proposed by West et al. [22] (| skewness | < 3, | kurtosis | < 8) and Hong et al. [23] (| skewness | < 2, | kurtosis | < 4), based on which normality was deemed to have been established. Table 1 shows the mean, standard deviation, item scale, skewness, kurtosis, and reliability of the collected data at each time-point. 

### 3.2. Correlations

There were statistically significant correlations among all variables at each time-point (*p* < 0.05). However, there were weak correlations between PE achievement and both social relationships (0.299–0.371) and sense of community (0.189–0.234) at each time-point. Nevertheless, there was a strong correlation between social relationships and sense of community (0.692–0.749). Table 2 lists the correlation coefficients for each time-point. 

### 3.3. Nested Model Comparisons

Sixteen competitive models were established to verify the measurement, path coefficient, and error covariance invariance. Model 1 was the basic model without any constraints, and Models 2–4 were used to verify measurement invariance. Models 5–13 were used to verify path invariance, and Models 5–7 had cross-lagged coefficients with invariance constraints. Models 14–16 were used to verify the error covariance invariance. A structural equation model is deemed to have a good fit when the CIF, an incremental fit index, is 0.90 or higher, and the absolute fit indices, RMSEA and SRMR, are ≤ 0.05 or lower [24,25]. Models 1–16 satisfied these criteria. 

For an autoregressive cross-lagged model, when the sample size is appropriate (N > 300) and equal across time-points, measurement, autoregressive coefficient, and error covariance invariance are established if CFI ≤ 0.010, RMSEA ≤ 0.015, and SRMR ≤ 0.010 between competitive models [26,27]. The ΔCFI, ΔRMSEA, and ΔSRMR between our models were smaller than 0.010; therefore, the above invariances were established. Table 3 presents the fit indices for each model and the differences between them.

### 3.4. Structural Regression Analysis

**Figure 2:**
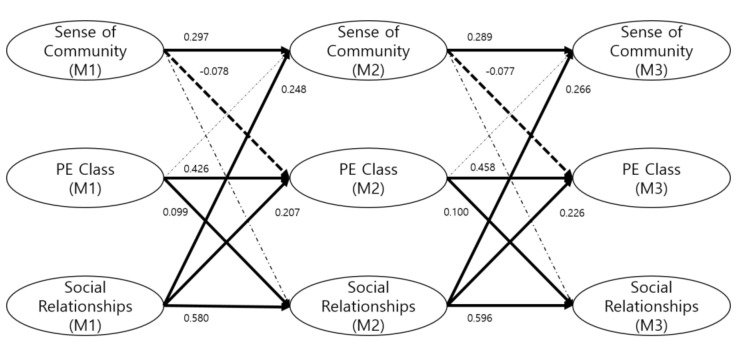
Results of the research model.

The autoregressive coefficients among PE achievement, social relationships, and sense of community were measured at the three time-points. As shown in Figure 2, PE achievement at an earlier time-point had a statistically significant impact on PE achievement at a later time-point (β = 0.426, β = 0.458, *p* < 0.01). Moreover, social relationships at an earlier time-point had a statistically significant impact on social relationships at a later time-point (β = 0.580, β = 0.596, *p* < 0.01). Similarly, sense of community at an earlier time-point also had a statistically significant impact on sense of community at a later time-point (β = 0.297, β = 0.289, *p* < 0.01). These results show that PE achievement, social relationships, and sense of community were stably predicted despite changes over three years.

Regarding study question 1, we examined the causation between PE achievement and social relationships over the course of the regression. As shown in Figure 2, PE achievement at M1 had a statistically significant positive effect on social relationships at M2 and M3 (β = 0.099, β = 0.100, *p* < 0.01). Similarly, social relationships at M1 also had a statistically significant positive effect on PE achievement at M2 and M3 (β = 0.207, β = 0.226, *p* < 0.01). These results show that PE achievement improves social relationships, and social relationships help students make accomplishments in PE. Further, these results support the prediction that students with higher PE achievement at M1 will have better social relationships at M2 and M3, and those who have better social relationships at M1 will have higher PE achievement at M2 and M3. 

Regarding study question 2, PE achievement at M1 did not have a statistically significant effect on sense of community at M2 or M3. However, sense of community at M1 had a statistically significant negative effect on PE achievement at M2 and M3 (β = –0.078, β = –0.077, *p* < 0.01). These results suggest that PE achievement does not foster a sense of community; moreover, a sense of community lowers PE achievement. These results support the prediction that students with a high sense of community at M1 will have lower PE achievement at later time-points. 

Regarding study question 3, social relationships at M1 had a statistically significant positive effect on sense of community at M2 and M3 (β = 0.248, β = 0.266, *p* < 0.01). However, sense of community at M1 did not affect social relationships at M2 and M3. These results suggest that while social relationships can foster a sense of community, a sense of community does not improve social relationships. These results support the prediction that students with better social relationships at M1 will have a higher sense of community at later time-points.

Additionally, we examined the reciprocal causation among PE achievement, social relationships, and sense of community. There was partial reciprocal causation, that is, a cause-effect relationship between PE achievement and social relationships, between social relationships and PE achievement, between the sense of community and PE achievement, and between social relationships and sense of community. Table 4 and Figure 2 show the results of path analysis.

## 4. Discussion

Generally, PE researchers argue that PE can promote students’ social health [1,2,9,28]. However, there have been limitations in substantiating the causal relationship between PE and social health [2], as generalizing research findings has been difficult because these studies did not analyze large-scale, long-term data [10]. Thus, we aimed to analyze the causal relationship between PE and social health using a large-scale, long-term dataset. More specifically, we explored the causal relationships between PE achievement and social relationships (parents, peers, and teachers), PE achievement and sense of community, and social relationships and sense of community.

### 4.1. PE Achievement and Social Relationships

In our analysis, there was a reciprocal causation between PE achievement and social relationships. In other words, not only does PE unilaterally improve social relationships but social relationships also boost PE achievement. Such a reciprocal causal relationship between PE achievement and social relationships has also been documented in previous studies [9,28]. Based on the standardization coefficients between PE achievement and social relationships, the impact of PE achievement on social relationships was greater than the impact of social relationships on PE achievement. 

Bailey [28] stated that the purpose of physical activities in PE is to realize one’s potential [28]. This suggests that simply participating in PE alone cannot guarantee any educational effects. Regarding the mechanism through which PE improves students’ social relationships, many scholars have stated that this is not a guaranteed outcome [29] but is dependent on the instructor [1,2,30,31]. PE teachers’ perceptions of the importance of interaction [1] and the inherent social-educational curriculum in PE classes improve social relationships among students [2]. Both PE teachers’ perceptions and PE curricula are directly relevant to the PE teachers, that is, PE teachers’ determination in PE classes is associated with the improvement of students’ social relationships. In this study, PE achievement influenced social relationships at all time-points, indicating that students’ perceived PE achievement predicted changes in their social relationships.

Moreover, social relationships also influenced PE achievement at all time-points. A social relationship refers to a meaningful relationship formed based on interactions with other people [5]. Bailey [28] stated that parents, teachers, and friends serve as a bridge connecting students to PE, which suggests that social relationships are associated with the quality of PE participation. Thus, programs to enhance social relationships should be incorporated into PE to encourage students to further engage in their PE classes, as improvement of social relationships predicts PE achievement. In this regard, the results of this study support the need for programs that enhance social relationships in PE classes. We confirmed that PE achievement and social relationships continue to influence each other in a reciprocal relationship over time. Thus, PE teachers should design their curriculum and programs such that PE achievement and social relationship improvement can be appropriately controlled in the class to readily achieve the PE goals.

### 4.2. PE and Sense of Community

Sense of community in this study is similar to the concept of social responsibility examined in previous research [5]. Previous studies have reported that PE is positively associated with prosocial behaviors, such as a sense of community [9,32], and has a positive effect on social responsibility [33]. Parents also expect PE to contribute to boosting social responsibility and teamwork for their children [34]. The reason for the positive effect of PE on sense of community is because one of the traditional goals of PE is to acquire prosocial behaviors, and for this reason, behaviors related to sense of community have been emphasized as desirable [9]. However, PE does not always facilitate students’ acquisition of prosocial behaviors, such as a sense of community [35]. Specifically, participation in PE does not guarantee students will learn prosocial behaviors, and some students may actually perceive PE as a negative experience [5]. In this study, PE achievement did not influence sense of community. Sense of community is known to be fostered by the process of collaboration in which more skilled students teach and encourage less skilled students [36]. Therefore, we can speculate that the PE offered in Korean middle schools lacks collaborative programs that cultivate a sense of community.

The results of this study sound an alarm regarding ascribing too much meaning to PE beyond its role as a background for activities to learn prosocial behaviors. PE teachers play an important role in fostering an educational environment in which students learn a sense of community [5]. At the same time, they need to be equipped with teaching skills with intentional and systematic methodologies to ensure positive outcomes [37]. Students’ acquisition of social responsibility, such as a sense of community, is linked to the instructor’s expertise (ability to foster an educational environment and systematic teaching skills). Students’ PE achievement varies depending on the class quality as determined by the PE teacher’s expertise. Moreover, social skills, such as a sense of community, are also critical in other areas of life. Considering that accomplishment in PE can be transferred to other areas of life [38], developing PE teachers’ instructional competency to foster a sense of community among students is also crucial.

Traditionally, PE scholars have focused on substantiating the effects of PE; therefore, they mostly examined the unidirectional effect of PE and sports on sense of community [9]. However, this study observed the relationship between PE achievement and sense of community over several time-points. While PE achievement did not affect sense of community, the latter had a negative effect on PE achievement. Moreover, PE differs according to various historical, social, and political contexts and is socially structured [39]. The results of this study also reflect the unique social context of the Korean society. We speculate that these results were influenced by Korean students’ tendencies to place low importance on PE. This is because while college entrance is a socially critical phenomenon in Korea, PE achievement is not a factor considered in college entrance [7]. Therefore, this result may be attributable to the fact that Korean students’ passive attitudes during PE are actually perceived as them being responsible students.

### 4.3. Social Relationship and Sense of Community

The development of personal and social skills is a significant learning outcome, as it not only enables students to become successful learners but also successfully transition into adulthood [40]. In this regard, developing personal and social skills is a widely accepted goal in PE and sports worldwide. However, while evidence for the effects of PE and sports on the development of personal and social skills is growing, the terminology and method of use of personal and social skills are fragmented [5]. In other words, terms referring to similar concepts are used in different forms. The World Health Organization defines personal and social skills as psychosocial abilities or life skills [41]. Opstoel et al. [5] reviewed 88 studies pertinent to these life skills and classified them into 11 themes. Unfortunately, the association among these 11 themes has only been fragmentarily researched [9].

In this longitudinal study, at all time-points, social relationships had a positive effect on sense of community, while sense of community did not influence social relationships. This confirmed that the development of social relationships can predict the formation of a sense of community. Undoubtedly, one of the major goals of PE is to promote the development of students’ personal and social skills [41]. The findings of this study suggest that programs that promote various social relationships should be implemented in PE classes before programs that foster a sense of community. Notably, efforts to foster a sense of community may be ineffective without first promoting the development of social relationships.

### 4.4. Limitations

In this study, we used data collected from middle school students in Korea from 2013 to 2015. A high school period should definitely be included if the aim is to investigate longitudinal changes in adolescence. However, due to the unique situation surrounding the college entrance exam in Korea, PE in middle schools is starkly different from that in high schools. Therefore, there are limitations regarding generalizing the findings of this study pertaining to the causation of PE achievement, social relationships, and sense of community among the entire Korean adolescent population.

## 5. Conclusions

The purpose of this study was to verify the traditional belief that physical education classes have a positive relationship with social health. As indicators of social health, students’ social relationships (with their parents, friends, and teachers) and sense of community were checked. The following conclusions were drawn. First, there was reciprocal causation between PE achievement and social relationships. To improve social relationships, PE classes should offer a system through which students can frequently check their performance, and the PE curriculum should be designed to promote social relationships, to boost the quality of PE participation. Second, there was a causal relationship between PE achievement and sense of community, where the latter had a negative effect on PE achievement. This suggests that cooperation-promoting programs should be considered for Korean middle school PE classes to foster a sense of community among students. Third, there was a unidirectional causal relationship between social relationships and sense of community. This suggests that an appropriate hierarchy must be established when designing PE programs.

Additionally, we suggest subsequent studies to follow up on the negative effect of sense of community on PE achievement observed in this study. Researchers should identify whether this is a unique phenomenon observed among Korean middle school students, a phenomenon evident in all Korean adolescent populations—including high school students—or common worldwide.

## Figures and Tables

**Table 1:** Descriptive statistics for the main variables.

		Mean	Standard Deviation	Range	Skewness	Kurtosis	Cronbach α
PE achievement score	M1	4.32	2.41	1–8	0.20	−1.27	0.728
	M2	3.84	2.42		0.45	−1.11	0.745
	M3	3.37	2.37		0.74	−0.76	0.713
PE achievement—subjective evaluation	M1	2.72	1.13	1–5	0.12	−0.67	0.728
	M2	2.57	1.16		0.33	−0.61	0.745
	M3	2.41	1.11		0.41	−0.52	0.713
Relationship with parents	M1	1.76	0.53	1–4	0.14	−0.11	0.774
	M2	1.78	0.54		0.27	0.31	0.734
	M3	1.81	0.51		0.04	−0.31	0.727
Peer relationship	M1	1.86	0.47	1–4	0.13	0.89	0.716
	M2	1.85	0.45		−0.18	0.09	0.721
	M3	1.83	0.44		−0.09	0.58	0.722
Relationship with teachers	M1	2.02	0.64	1–4	0.28	0.22	0.843
	M2	2.01	0.62		0.22	0.21	0.850
	M3	1.97	0.60		0.13	0.10	0.848
Sense of community	M1	2.00	0.58	1–4	0.12	0.29	0.802
	M2	2.04	0.54		−0.04	0.37	0.785
	M3	1.99	0.53		−0.07	0.24	0.783

**Table 2:** Correlations among the main variables.

	1	2	3	4	5	6	7	8
1. PE Class (M1)								
2. PE Class (M2)	0.441 **							
3. PE Class (M3)	0.401 **	0.528 **						
4. Social Relationship (M1)	0.299 **	0.308 **	0.202 **					
5. Social Relationship (M2)	0.289 **	0.321 **	0.280 **	0.563 **				
6. Social Relationship (M3)	0.274 **	0.259 **	0.371 **	0.472 **	0.643 **			
7. Sense of Community (M1)	0.234 **	0.160 **	0.129 **	0.692 **	0.373 **	0.341 **		
8. Sense of Community (M2)	0.154 **	0.189 **	0.174 **	0.468 **	0.702 **	0.485 **	0.447 **	
9. Sense of Community (M3)	0.195 **	0.198 **	0.214 **	0.395 **	0.450 **	0.749 **	0.397 **	0.503 **

** *p* < 0.05.

**Table 3:** Fit indices for the nested model comparisons.

Model	χ^2^	*df*	CFI	RMSEA (90% CI)	SRMR	ΔCFI	ΔRMSEA	ΔSRMR
Model 1	1138.118	270	0.967	0.037 (0.035, 0.039)	0.0364	-	-	-
Model 2	1142.869	272	0.967	0.037 (0.035, 0.039)	0.0364	0.000	0.000	0.000
Model 3	1152.804	278	0.966	0.036 (0.034,0.039)	0.0365	0.001	0.001	0.001
Model 4	1161.517	282	0.966	0.036 (0.034, 0.038)	0.0364	0.000	0.000	0.001
Model 5	1167.91	283	0.966	0.036 (0.034, 0.038)	0.0364	0.000	0.000	0.000
Model 6	1173.057	284	0.966	0.036 (0.034, 0.038)	0.0363	0.000	0.000	0.001
Model 7	1180.349	285	0.966	0.036 (0.034, 0.039)	0.0367	0.000	0.000	0.004
Model 8	1185.796	286	0.966	0.036 (0.034, 0.039)	0.0368	0.000	0.000	0.001
Model 9	1188.742	287	0.965	0.036 (0.034, 0.039)	0.0368	0.001	0.000	0.000
Model 10	1200.227	288	0.965	0.037 (0.034, 0.039)	0.0370	0.000	0.001	0.002
Model 11	1200.271	289	0.965	0.036 (0.034, 0.039)	0.0370	0.000	0.001	0.000
Model 12	1200.342	290	0.965	0.036 (0.034, 0.038)	0.0371	0.000	0.000	0.001
Model 13	1209.031	291	0.965	0.036 (0.034, 0.039)	0.0373	0.000	0.000	0.002
Model 14	1215.123	292	0.965	0.036 (0.034, 0.039)	0.0375	0.000	0.000	0.002
Model 15	1217.891	293	0.965	0.036 (0.034, 0.039)	0.0374	0.000	0.000	0.001
Model 16	1217.919	294	0.965	0.036 (0.034, 0.038)	0.0374	0.000	0.000	0.000

**Table 4:** Structural regression analysis results for the final model.

Parameter			Estimate	*SE*	CR	Standardized	*p*
PE_M1	→	PE_M2	0.432	0.016	26.63	0.426	0.003 **
PE_M2	→	PE_M3	0.432	0.016	26.63	0.458	0.003 **
Social_M1	→	Social_M2	0.597	0.032	18.889	0.580	0.002 **
Social_M2	→	Social_M3	0.597	0.032	18.889	0.596	0.002 **
Comm_M1	→	Comm_M2	0.277	0.027	10.064	0.297	0.006 **
Comm_M2	→	Comm_M3	0.277	0.027	10.064	0.289	0.006 **
PE_M1	→	Social_M2	0.035	0.006	6.073	0.099	0.003 **
PE_M2	→	Social_M3	0.035	0.006	6.073	0.100	0.003 **
Social_M1	→	PE_M2	0.617	0.085	7.296	0.207	0.006 **
Social_M2	→	PE_M3	0.617	0.085	7.296	0.226	0.006 **
PE_M1	→	Comm_M2	0.008	0.003	2.204	0.036	0.079
PE_M2	→	Comm_M3	0.008	0.003	2.204	0.038	0.079
Comm_M1	→	PE_M2	−0.353	0.124	−2.857	−0.078	0.005 **
Comm_M2	→	PE_M3	−0.353	0.124	−2.857	−0.077	0.005 **
Social_M1	→	Comm_M2	0.151	0.018	8.19	0.248	0.002 **
Social_M2	→	Comm_M3	0.151	0.018	8.19	0.266	0.002 **
Comm_M1	→	Social_M2	0.017	0.046	0.377	0.011	0.682
Comm_M2	→	Social_M3	0.017	0.046	0.377	0.010	0.682

** *p* < 0.05.

## Data Availability

The datasets used and/or analyzed during this study are included in this published article.

## Appendix A

**PE achievement**

1. What was your grade in PE class last semester?

① ≥ 96 ② 95–90 ③ 89–85 ④ 84–80 ⑤ 79–75 ⑥ 74–70 ⑦ 69–65 ⑧ ≤ 64

2. How would you rate your performance in your PE class in the past semester compared to other students?

① Very good ② Good ③ So-So ④ Bad ⑤ Very bad

**Social relationship**

Please indicate the number that best represents your response to the following statements.

① Strongly agree ② Agree ③ Disagree ④ Strongly disagree

**Relationship with parents**

1. My parents consider me to be more important than other things (e.g., work).
2. My parents show interest in and ask about my school life.
3. My parents always ensure that I keep myself clean and my clothes and bedding are clean.
4. My parents provide me with proper care when I am really sick.

**Peer relationship**

1. I get along with other kids in my class.
2. I apologize first when I get into a fight with a friend.
3. When my tablemate forgets to bring their textbook or other supplies, I share mine.
4. My opinions or suggestions are well received by other children when we play or do group activities.

**Relationship with teachers**

1. I say “Hi” to my teachers when I encounter them.
2. I feel comfortable talking to my teachers.
3. I like bumping into my teachers outside the school.
4. My teachers are friendly with me.

**Sense of community**

1. I am willing to actively help my friends who are in trouble.
2. I am willing to give up my free time and volunteer at a welfare center on holidays.
3. I am willing to make donations to countries that are poorer than mine.
4. I am willing to actively participate in practices such as conserving goods, appropriately using recycling bins, and recycling products to protect the earth.
